# Supplementary material for: Crystal structures of the human neurokinin 1 receptor in complex with clinically used antagonists
Source: Nat Commun. 2019 Jan 3;10:17. doi: 10.1038/s41467-018-07939-8 (PMC6318301; doi:10.1038/s41467-018-07939-8)
Supplement: Supplementary file 1 — Supplementary Information [file 41467_2018_7939_MOESM1_ESM.pdf]

Supplementary Information

Crystal structures of the human neurokinin 1 receptor in complex with clinically used antagonists

J. Schöppe, J. Ehrenmann et al.

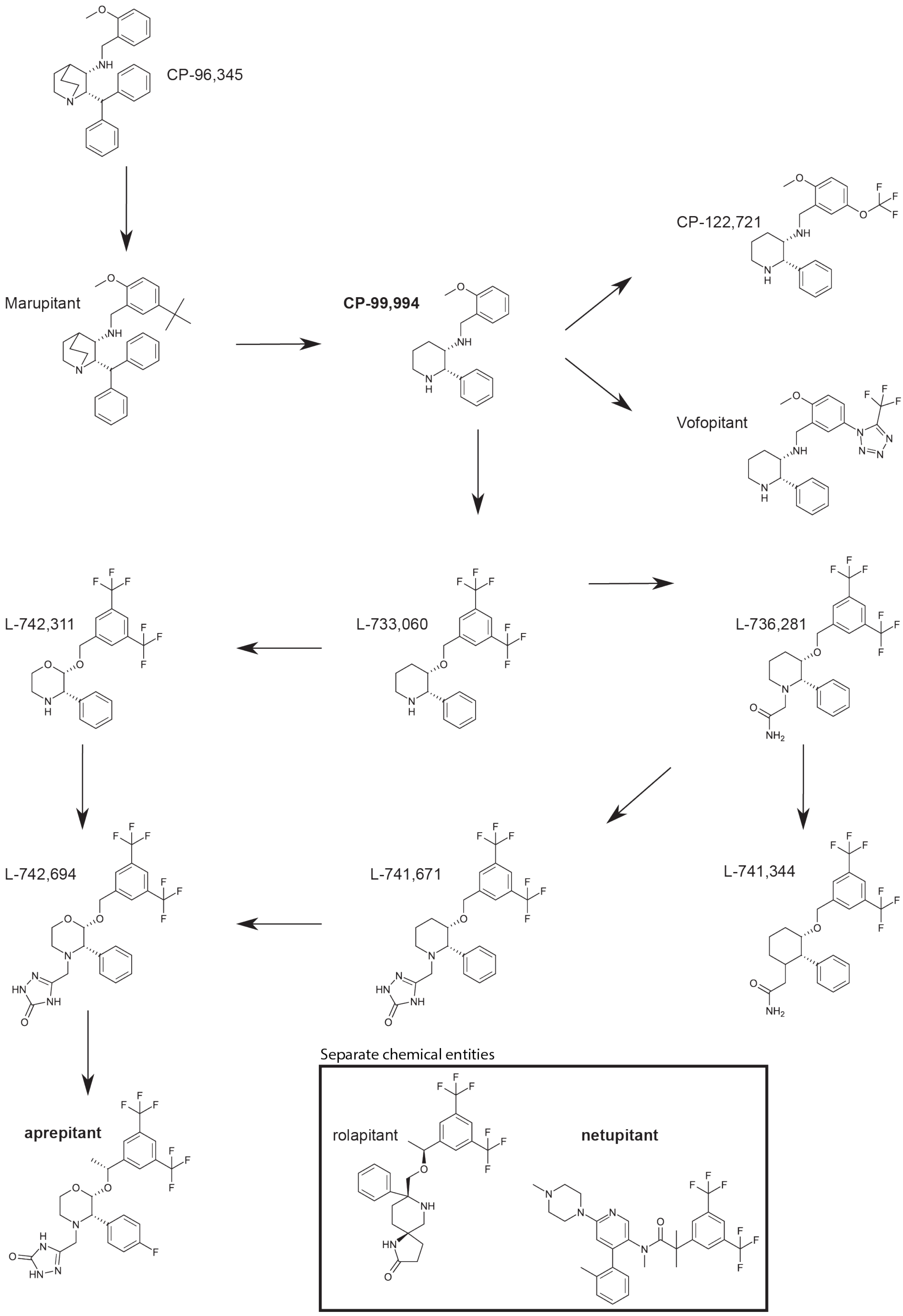

Supplementary Figure 1. Overview of non-peptide NK_1R antagonist development.

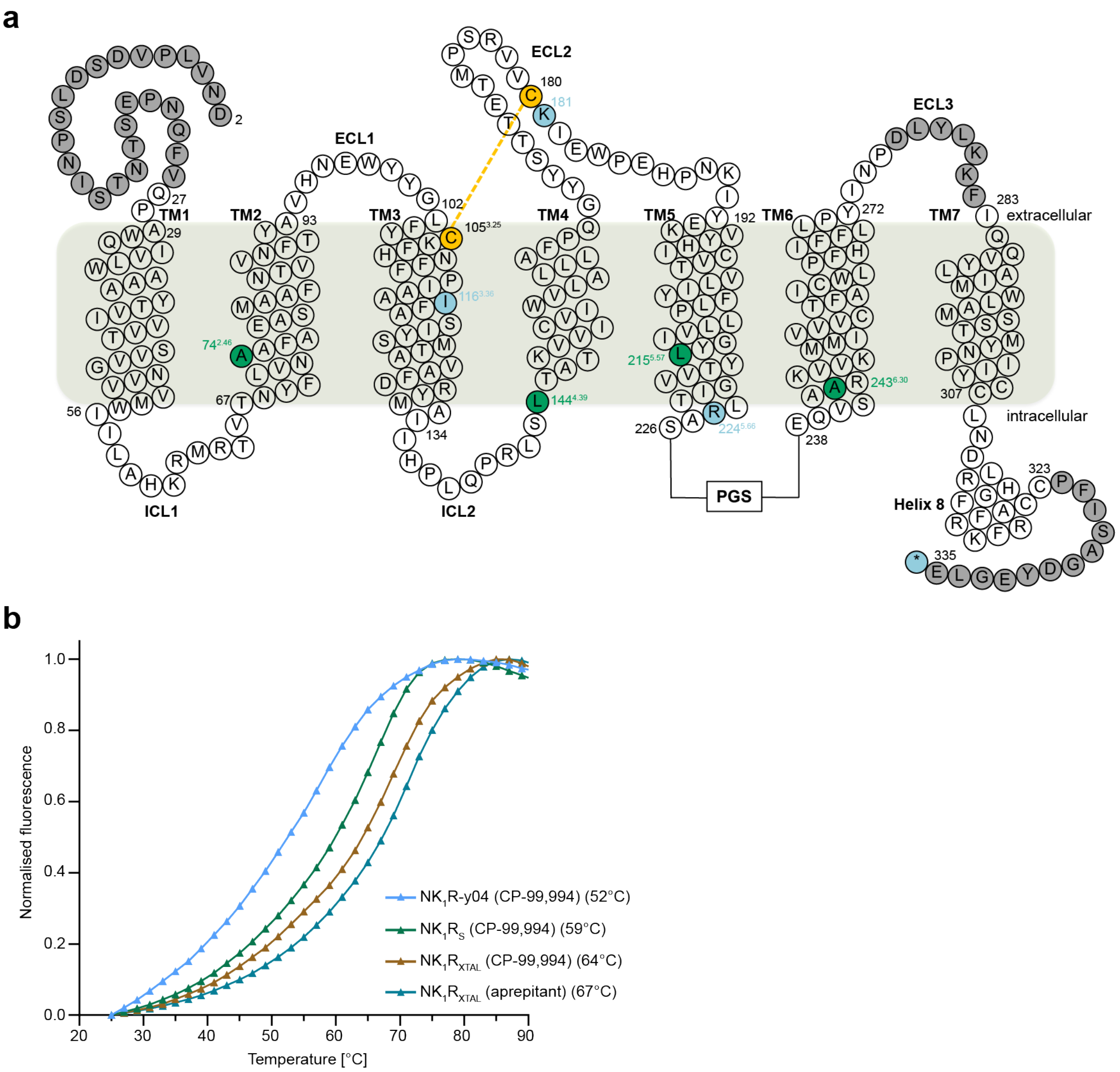

Supplementary Figure 2. Overview of the crystallised NK$_1$R construct and thermostability of NK1R mutants bound to CP-99,994 or aprepitant. (a) Snake plot of the crystallised NK$_1$R construct NK$_1$R$_{XTAL}$. Residues 336-407 at the receptor C-terminus were truncated and residues 227-237 of ICL3 were replaced by a PGS fusion. Mutations from directed evolution in *S. cerevisiae* and the additionally introduced thermostabilising mutations are highlighted in blue and green, respectively. The conserved disulfide bond is indicated by a dashed yellow line. The first and last residues of transmembrane helices I-VII (TM1-7) are labelled with the residue number. Residues which are not resolved in the crystal structure are shown in grey. (**b**) Thermostability assay (CPM) of the yeast-evolved NK$_1$R (NK$_1$R-y04) bound to CP-99,994, the further thermostabilised NK$_1$R (NK$_1$R$_S$) bound to CP-99,994 and the crystallisation construct NK$_1$R$_{XTAL}$ bound to CP-99,994 and aprepitant. The respective melting temperatures are indicated in parentheses.

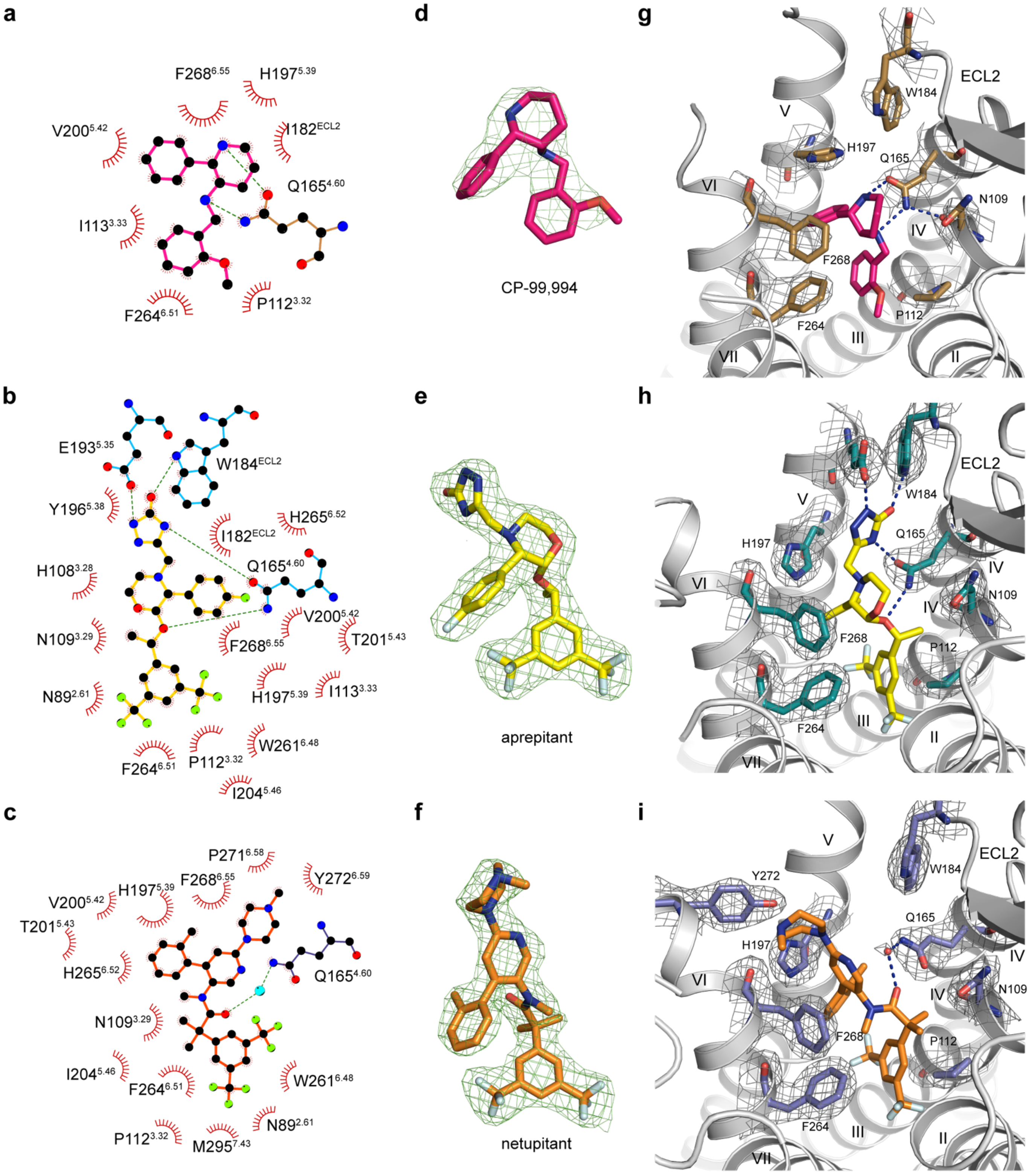

Supplementary Figure 3. Co-crystallised NK$_1$R antagonists and sidechain electron density within the orthosteric pocket. (**a-c**) Schematic representation of interactions between NK$_1$R and the antagonists CP-99,994 (**a**), aprepitant (**b**) and netupitant (**c**) analysed by LigPlot+ (Laskowski, R. A. *et al.* LigPlot+: multiple ligand–protein interaction diagrams for drug discovery. *J. Chem. Inf. Model.* **51**, 2778–2786 (2011)). The ordered water involved in netupitant binding is depicted as a light-blue sphere (**c**). Hydrogen bonds are indicated by dashed lines. (**d-f**)**,** The co-crystallised antagonists CP-99,994 (**d**), aprepitant (**e**) and netupitant (**f**) are shown as sticks. Oxygen, nitrogen and fluorine atoms of the ligands are highlighted in red, blue and grey, respectively. 2F$_o$-F$_c$ electron density maps of the ligands are shown in green mesh contoured at 1.0 σ. (**g-h**) 2F$_o$-F$_c$ electron density maps contoured at 1.0 σ for the

sidechains of key interaction residues within the orthosteric pocket of NK_1R structures solved in complex with CP-99,994 (**g**), aprepitant (**h**) and netupitant (**i**). Receptor and ligand representations are as in **Figure 2a-c**.

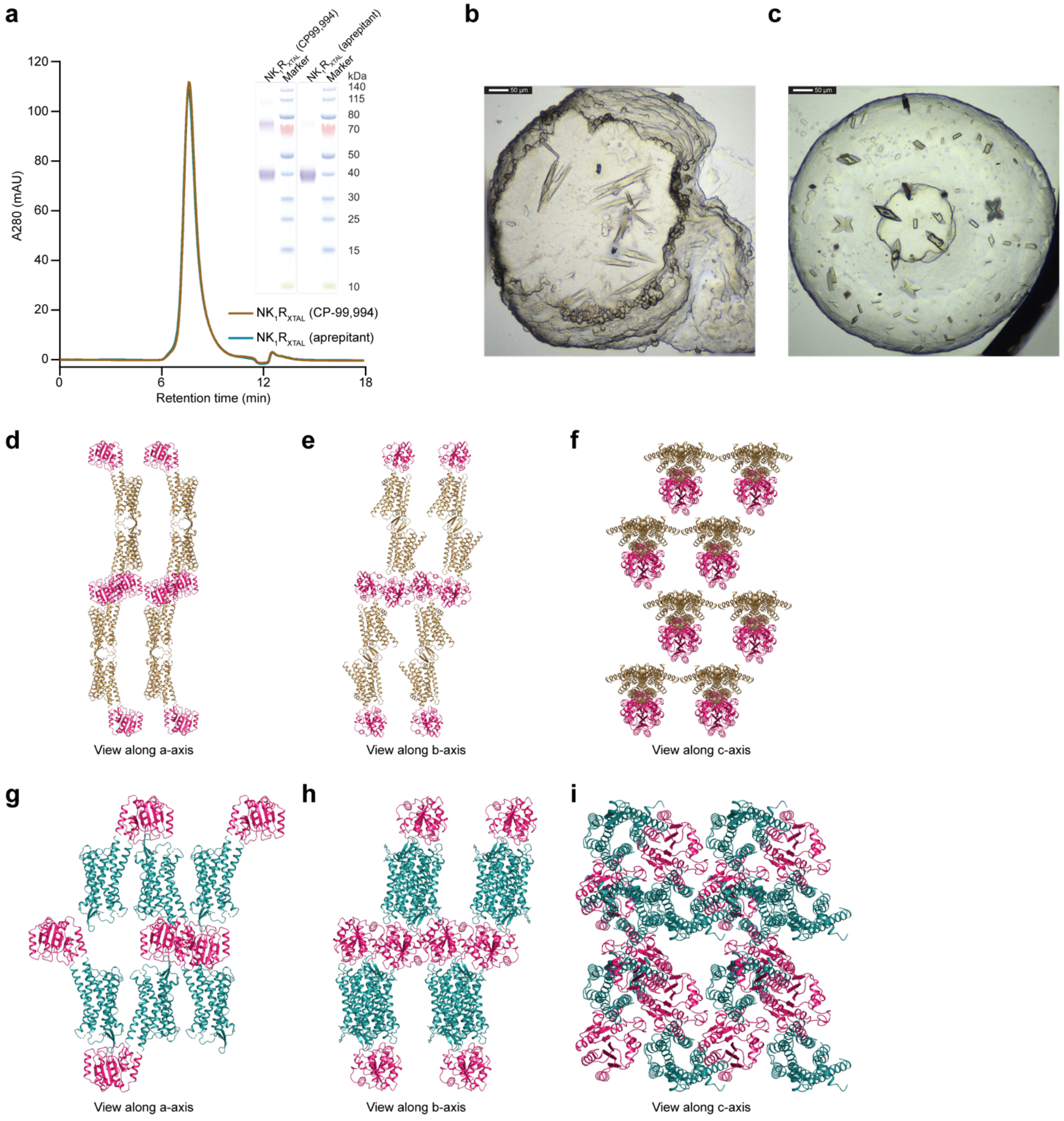

Supplementary Figure 4. NK$_1$R crystallisation. (**a**) Size exclusion profiles and SDS-PAGE gels of purified NK$_1$R$_{XTAL}$ in complex with CP-99,994 or aprepitant. (**b**) Bright-field image of NK$_1$R$_{XTAL}$:CP-99,994 complex crystals in lipidic cubic phase. (**c**) Bright-field image of NK$_1$R$_{XTAL}$:aprepitant complex crystals in lipidic cubic phase. (**d-i**) Packing of NK$_1$R$_{XTAL}$ crystals as views along axes a, b and c of the unit cell (PGS fusion shown in pink). NK$_1$R$_{XTAL}$:CP-99,994 (coloured in brown) crystallised in space group C222$_1$ (**d-f**). NK$_1$R$_{XTAL}$:aprepitant (coloured in turquois) crystallised in space group P2$_1$2$_1$2$_1$ (**g-i**).

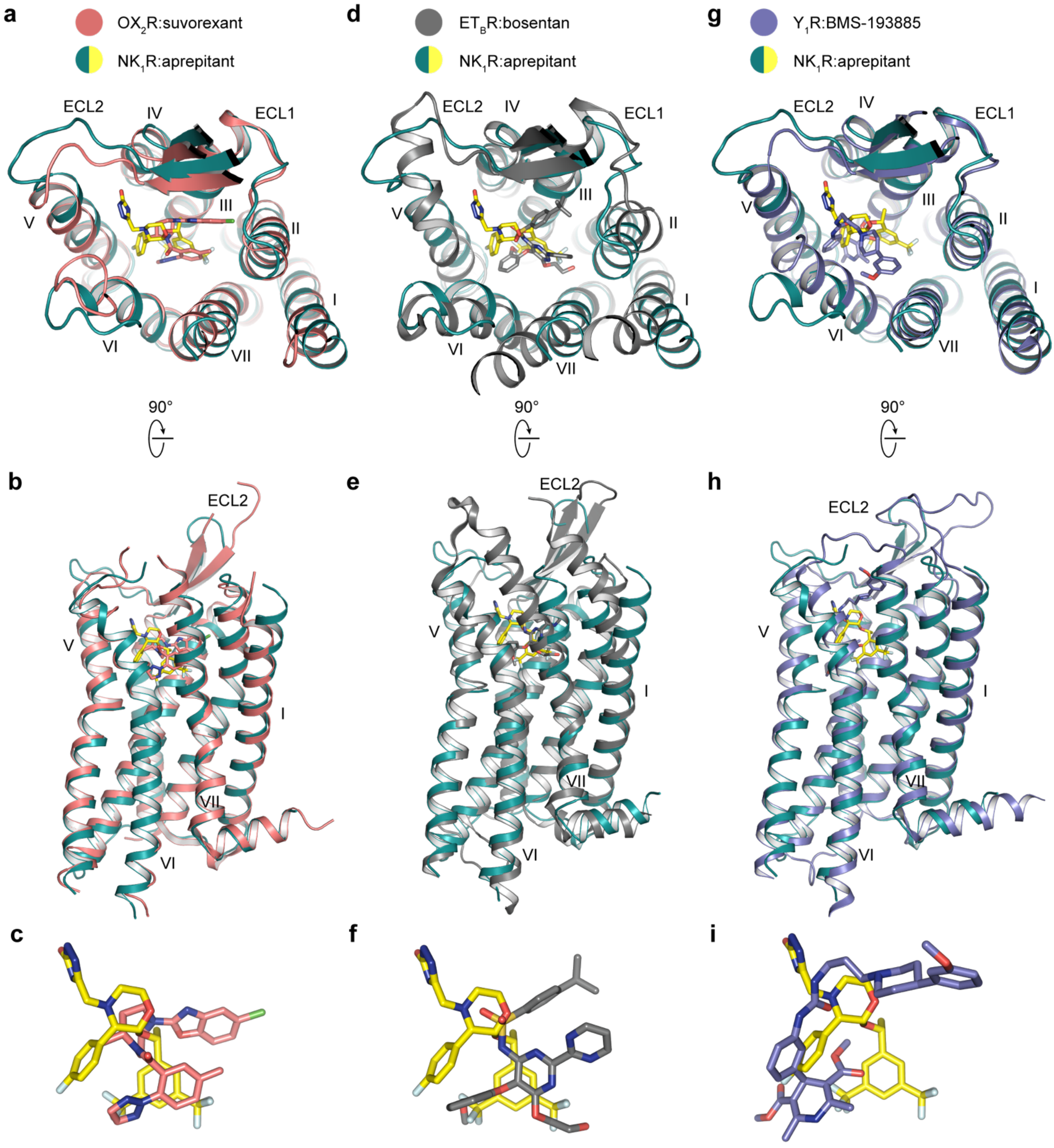

Supplementary Figure 5. Comparison of antagonist binding mode of NK_1R and other peptidergic GPCRs. (**a-c**) Overlay of the structures of NK_1R:aprepitant and OX_2R:suvorexant (PDB ID 4S0V) as viewed from the extracellular space (**a**), from the membrane plane (**b**) and the isolated antagonists viewed from helix VI-VII (**c**). **(d-f)** Overlay of the structures of NK_1R:aprepitant and ET_BR:bosentan (PDB ID 5XPR) as viewed from extracellular space (**d**), from the membrane plane (**e**) and the isolated antagonists viewed from helix VI-VII (**f**). (**g-i**) Overlay of the structures of NK_1R:aprepitant and Y_1R:BMS-193885 (PDB ID 5ZBH) as viewed from extracellular space (**g**), from the membrane (**h**) and the isolated antagonists viewed from helix VI-VII (**i**).

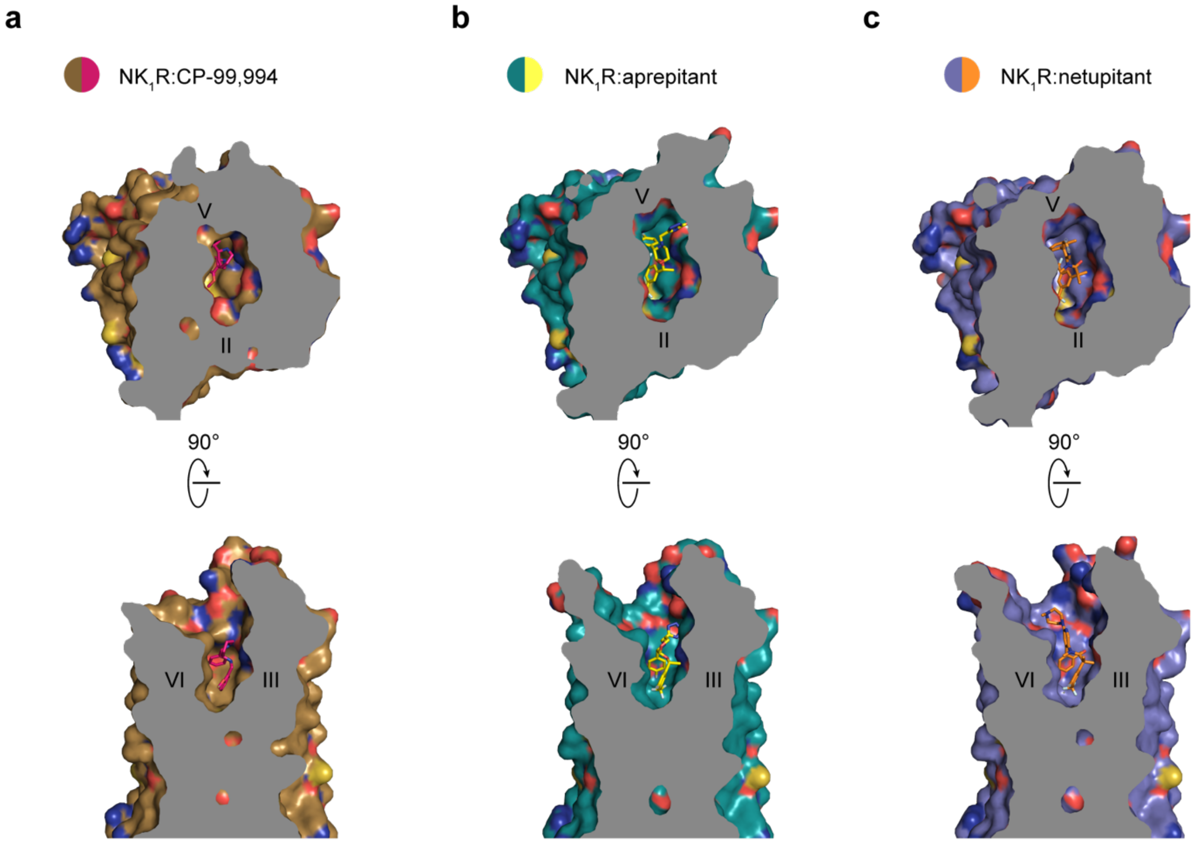

Supplementary Figure 6. Orthosteric binding pocket of NK$_1$R. (**a-c**) Cutaway view on the orthosteric binding pocket of NK$_1$R in complex with CP-99,994 (**a**), aprepitant (**b**) and netupitant (**c**) as viewed from the extracellular space (top) and parallel to the membrane plane from transmembrane helix II. The receptor:ligand complexes are coloured as in **Figure 1**. The position of transmembrane helices II and V (top) as well as III and VI (bottom) are indicated.

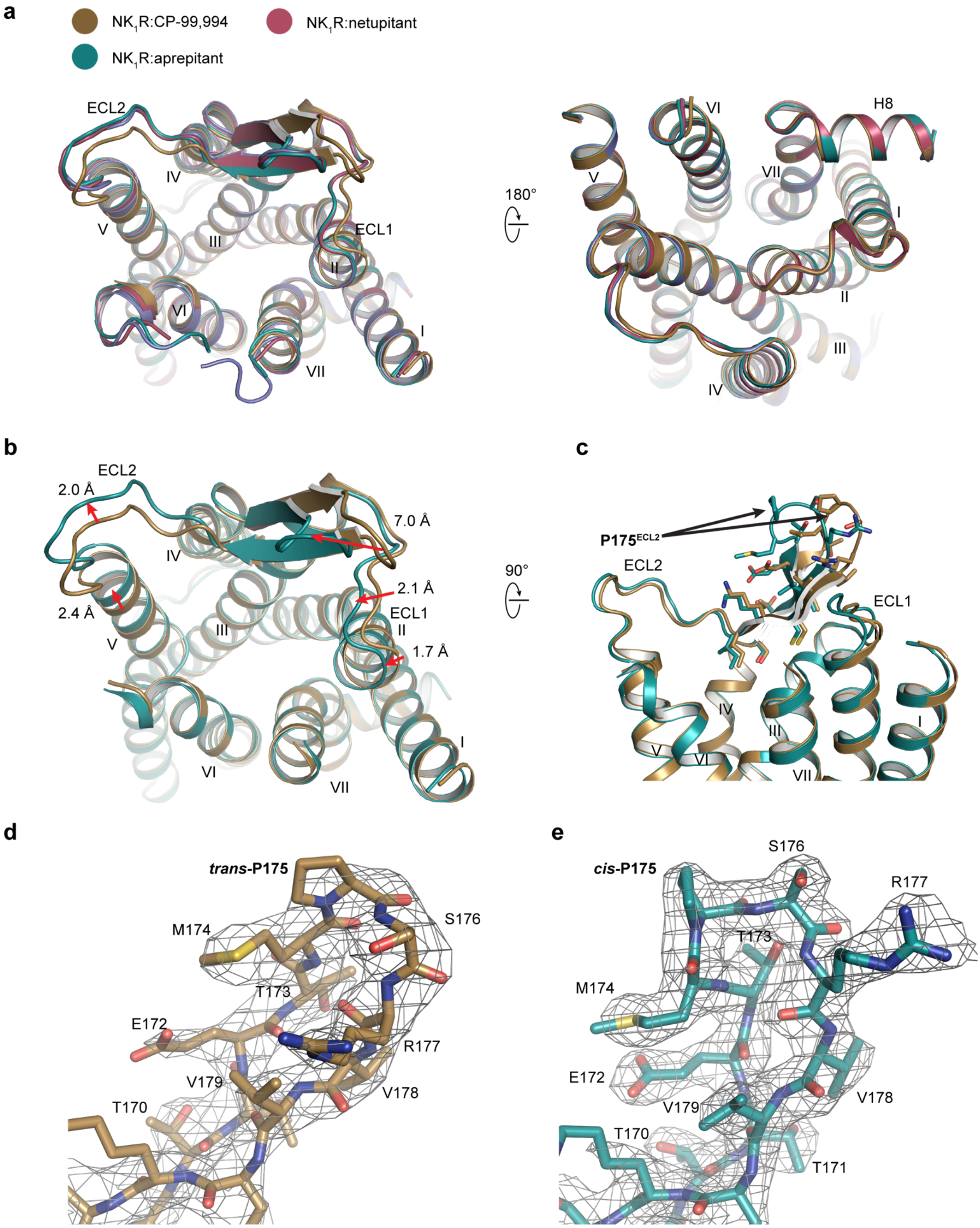

Supplementary Figure 7. Conformational differences between the structures of NK_1R in complex with different antagonists. (a) Superimposition of NK_1R in complex with CP-99,994, aprepitant and netupitant as viewed from the extracellular space and the cytoplasm. Receptors are coloured as in **Figure 1**. (**b**) Superimposition of the CP-99,994- and aprepitant-bound NK_1R as viewed from the extracellular space. Differences in receptor conformation are indicated by arrows. (**c**) Side-view on CP-

99,994- and aprepitant-bound NK_1R. Residues of the β-sheet and the β-hairpin of ECL2 are shown as sticks. The position of P175 in the β-hairpin of ECL2, which displays a different geometric isomerism in the two NK_1R conformations, is indicated by an arrow. (**d**) Close-up view on the β-sheet and the β-hairpin of ECL2 of the CP-99,994-bound NK_1R with the *trans*-P175 highlighted. (**e**) Close-up view on the β-sheet and the β-hairpin of ECL2 of the aprepitant-bound NK_1R with the *cis*-P175 highlighted.

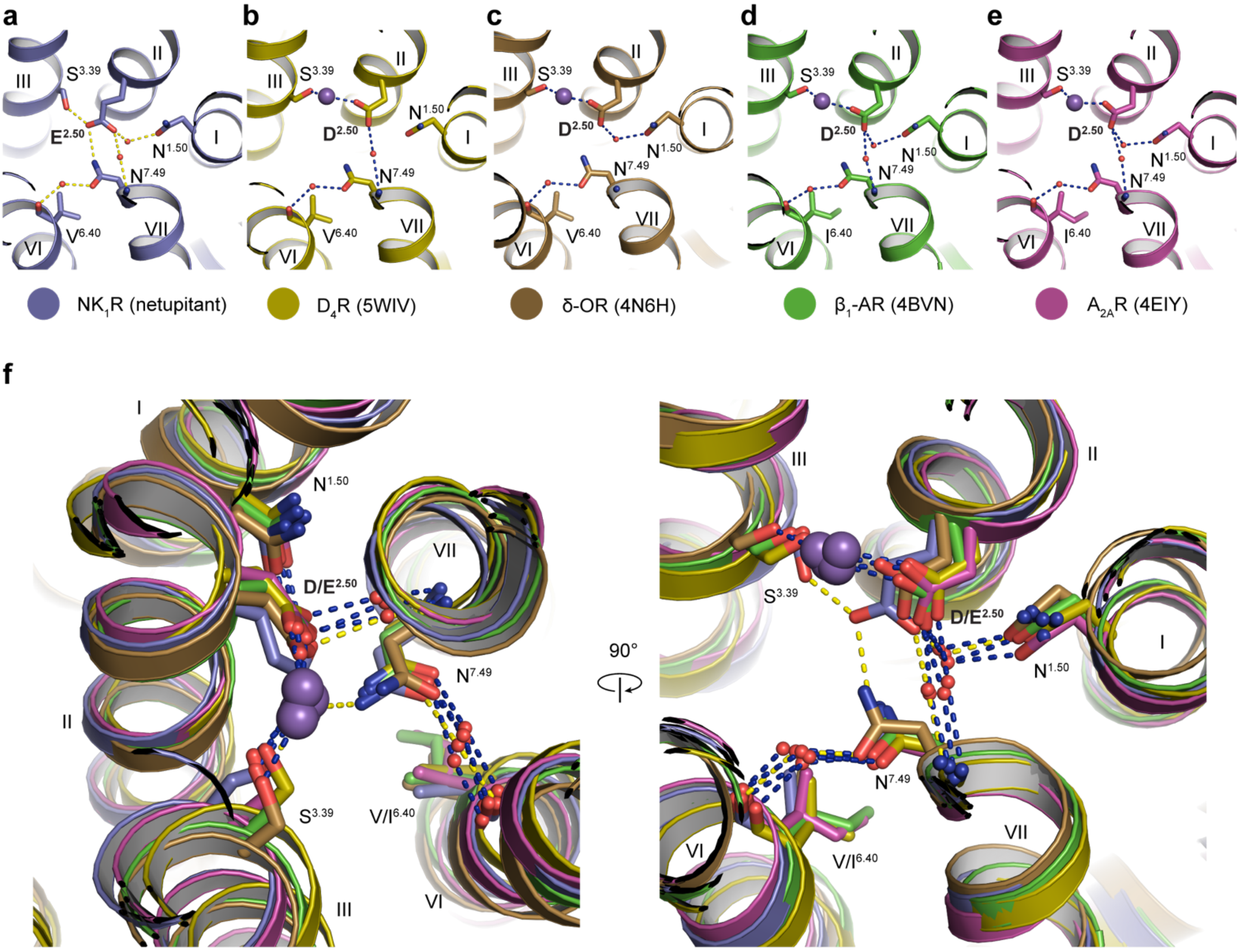

Supplementary Figure 8. Hydrogen bonding network in the transmembrane core of high-resolution GPCR crystal structures. (**a-e**) Extracellular view on the hydrogen bonding network in the transmembrane core of the netupitant-bound NK_1R (**a**), the dopamine D4 receptor (D_4R, PDB ID 5WIV) (**b**), the δ-opioid receptor (δ-OR, PDB ID 4N6H) (**c**), the β_1-adrenegic receptor (β_1-AR, PDB ID 4BVN) (**d**) and the adenosine A_{2A} receptor ($A_{2A}R$, PDB ID 4EIY) (**e**). Ordered waters are shown as red spheres, sodium ions are shown as purple spheres and hydrogen bonds are indicated by a dashed line. (**f**) Overlay of the hydrogen bonding networks from the receptors in (**a-e**). Hydrogen bonds in NK_1R are shown in yellow, hydrogen bonds in the other receptors are shown in blue.

Supplementary Table 1 Binding of antagonists to wild-type and mutated NK_1R variants.

construct	K_i (nM)			surface expression (% of WT)	n
	CP-99,994	aprepitant	netupitant		
wt	0.58±0.1	0.97±0.18	0.92±0.19	100	5
Q165A	164.1±52.0	4.76±0.75	4.76±0.77	120±7	5
Q165E	55.2±17.3	2.47±1.19	2.04±0.33	90±4	5
Q165D	*n.b.*	0.66±0.54	3.44±0.8	73±7	3
E193A	1.86±0.40	0.69±0.18	0.76±0.19	91±4	5
E193K	1.86±0.50	1.22±0.21	1.09±0.17	75±5	5
H197A	2.01±0.33	1.56±0.4	9.17±2.46	90±3	5
H197F	0.98±0.33	0.21±0.02	1.36±0.52	74±7	5
F264W	10.6±1.7	2.92±0.51	3.87±0.33	107±6	5
NK_1R-y04	2.79±0.36	0.74±0.31	0.86±0.11	46±7	3
NK_1R_{XTAL}	4.21±0.43	10.1±0.6	14.9±0.2	86±7	3

Whole-cell competition binding of SP-HL_{488} to HEK293T cells expressing wild-type and mutated NK_1R variants in the presence of different concentrations of unlabelled antagonists as a competitor. Data are shown as mean values ± SEM from 3-5 independent experiments performed in duplicate. *n.b.,* no binding.

Supplementary Table 2 Primers used for construct generation

Primer	Sequence (5’-to-3’)
NK_1R crystallisation construct generation	
NK1R-y04_L74A_for	CTATTTTCTGGTGAACGCCGCCTTCGCGGAGGCC
NK1R-y04_L74A_rev	GCCTCCGCGAAGGCGGCGTTCACCAGAAAATAGTTCGTC
NK1R-y04_A144L_for	GCCCCGGCTGTCACTGACAGCCACCAAAGTGGTC
NK1R-y04_A144L_rev	CTTTGGTGGCTGTCAGTGACAGCCGGGGCTGG
NK1R-y04_A215L_for	GGTGATTGGCTATCTGTACACCGTAGTGGGAATCAC
NK1R-y04_A215L_rev	CCCACTACGGTGTACAGATAGCCAATCACCAGCAGG
NK1R-y04_K243A_for	GAGCAAGTCTCTGCCGCCCGCAAGGTGGTCAAAATGATG
NK1R-y04_K243A _rev	GACCACCTTGCGGGCGGCAGAGACTTGCTCGTG
NK1R-y04_226_PGS_rev	GGACTCGTTCCAGAAGGAGCAGTCGATACCACTGGCCCGTAGTGTGATTC
NK1R-y04_238_PGS_for	AACTGCAAGAAACGCGCTATGTCCTTCTCCGAGCAAGTCTCTGCCGCC
NK1R-y04_1_pFL_for	TTGGAGGTGCTGTTCCAGGGTCCCATGGATAACGTCCTCCCG
NK1R-y04_335_pFL_rev	TGTGGACACGGCGGTGACCAGCACTTATTATTCCAGCCCCTCATAGTC
NK_1R constructs generation for antagonist binding affinity measurements	
NK1R_pcDNA3.1_for	AGACTGGGCAAGCCTGGGCTGGGTGATATCGATAACGTCCTCCCGGTG
NK1R_pcDNA3.1_rev	ACTCGAGCGGCCGCCACTGTGCTGGATTTAGGAGAGCACATTGGAGGAG
NK1R_165_MUT_rev	GGGGAAGGCCAGCAGGAGAGCCAG
NK1R_Q165A_for	CCTGGCTCTCCTGCTGGCCTTCCCCGCCGGCTACTACTCAACCACAGAG
NK1R_Q165E_for	CCTGGCTCTCCTGCTGGCCTTCCCCGAGGGCTACTACTCAACCACAGAG
NK1R_Q165D_for	CCTGGCTCTCCTGCTGGCCTTCCCCGACGGCTACTACTCAACCACAGAG
NK1R_193_MUT_rev	ATAAATCTTGTTCGGATGCTCTGGCCATTCG
NK1R_E193A_for	CCAGAGCATCCGAACAAGATTTATGCCAAAGTGTACCACATCTGTGTGAC
NK1R_E193K_for	CCAGAGCATCCGAACAAGATTTATAAGAAAGTGTACCACATCTGTGTGAC
NK1R_197_MUT_rev	GTACACTTTCTCATAAATCTTGTTCGGATGCTCTG
NK1R_H197A_for	GAACAAGATTTATGAGAAAGTGTACGCCATCTGTGTGACTGTGCTGATC
NK1R_H197F_for	GAACAAGATTTATGAGAAAGTGTACTTCATCTGTGTGACTGTGCTGATC
NK1R_264_MUT_rev	GGGCAGCCAGCAGATGGCGAAGGTG
NK1R_F264W_for	GCACCTTCGCCATCTGCTGGCTGCCCTGGCACATCTTCTTCCTCCTGCC
